# Demilt Dispensary, New York

**Published:** 1856-05

**Authors:** 


*Demilt Dispensary, New York.*—At the fifth anniversary meeting held lately, the Annual Report was read by the Secretary, Mr. Asahel Green, from which we learn that the whole number of patients treated by the several physicians during the past year, has been 20,004—males, 8,542; females, 11,462. Received at the Dispensary, 16,377; visited at their dwellings, 3,627; 7613, rather more than one-third of the whole number, were children. Of the 20,000, 7305 were natives. The results of treatment during the year have been that 116 had been sent to hospital, 195 had died, 19,693 had been discharged cured.—*Ibid.*

